# Association of food groups with depression and anxiety disorders

**DOI:** 10.1007/s00394-019-01943-4

**Published:** 2019-04-03

**Authors:** Deborah Gibson-Smith, Mariska Bot, Ingeborg A. Brouwer, Marjolein Visser, Erik J. Giltay, Brenda W. J. H. Penninx

**Affiliations:** 1 Department of Psychiatry, Amsterdam Public Health Research Institute, Amsterdam UMC, Amsterdam, The Netherlands; 2 Department of Health Sciences, Faculty of Science, Amsterdam Public Health Research Institute, Vrije Universiteit Amsterdam, Amsterdam, The Netherlands; 3 Department of Psychiatry, Leiden University Medical Center, Leiden, The Netherlands

**Keywords:** Mediterranean diet, Depression, Anxiety, Diet quality

## Abstract

**Purpose:**

Adherence to the Mediterranean diet has been associated with fewer depressive symptoms, however, it is unknown whether this is attributed to some or to all components. We examined the association between the individual food groups of the Mediterranean Diet Score (MDS), in isolation and in combination, with depression and anxiety (symptom severity and diagnosis).

**Methods:**

Data from 1634 adults were available from the Netherlands Study of Depression and Anxiety. Eleven energy-adjusted food groups were created from a 238-item food frequency questionnaire. In regression analysis, these were associated in isolation and combination with (1) depressive and anxiety disorders (established with the Composite International Diagnostic Interview) (current disorder *n* = 414), and (2) depression and anxiety severity [measured with the Inventory of Depressive Symptomatology (IDS), the Beck Anxiety Inventory (BAI) and the Fear Questionnaire (FEAR)].

**Results:**

Overall, the MDS score shows the strongest relationships with depression/anxiety [Diagnosis: odds ratio (OR) 0.77 per SD, 95% confidence interval (95% CI) 0.66–0.90, IDS: standardised betas (*β*) − 0.13, 95% CI − 0.18, − 0.08] and anxiety (BAI: *β* − 0.11, 95% CI − 0.16, − 0.06, FEAR: *β* − 0.08, 95% CI − 0.13, − 0.03). Greater consumption of non-refined grains and vegetables was associated with lower depression and anxiety severity, whilst being a non-drinker was associated with higher symptom severity. Higher fruit and vegetable intake was associated with lower fear severity. Non-refined grain consumption was associated with lower odds and being a non-drinker with greater odds of current depression/anxiety disorders compared to healthy controls, these associations persisted after adjustment for other food groups (OR 0.82 per SD, 95% CI 0.71–0.96, OR 1.26 per SD 95% CI 1.08–1.46).

**Conclusion:**

We can conclude that non-refined grains, vegetables and alcohol intake appeared to be the driving variables for the associated the total MDS score and depression/anxiety. However, the combined effect of the whole diet remains important for mental health. It should be explored whether an increase consumption of non-refined grains and vegetables may help to prevent or reduce depression and anxiety.

**Electronic supplementary material:**

The online version of this article (10.1007/s00394-019-01943-4) contains supplementary material, which is available to authorized users.

## Introduction

The 2013 Global Burden of Disease report identified that, in both developing and developed countries, major depressive disorder (MDD) now ranks as the second highest cause of years of life lost due to disability (YLD) [1]. Depression is an important public health problem and is estimated to affect more than 300 million people worldwide [2]. Furthermore, depression is frequently comorbid with anxiety disorders [3] which also represents a large burden to society as it is the sixth leading cause of disability in terms of YLDs [4].

There are indications that a healthy diet may play a protective role in the development, progression and treatment of depression. Meta-analyses of cross-sectional and longitudinal observational studies have shown that adherence to a healthy diet is inversely associated with the severity of depressive symptoms [5–8]. One of the most frequently used measure of a healthy diet is the Mediterranean diet score (MDS) [9]. The MDS combines the intake of 11 food groups into a summary score reflecting the level of adherence to a Mediterranean diet. Two meta-analysis found that Mediterranean diet score is more strongly associated with depressive symptoms compared to other dietary scores [6, 7]. Furthermore, a recent randomised controlled trial in 152 depressed patients indicates that adherence to a Mediterranean diet supplemented with fish oils can reduce depressive symptoms [10].

Analysing the overall dietary pattern, as these prior studies have done, has the benefit of evaluating the potentially synergistic effect of different food groups combined. However, studies focusing on diet quality score also have limitations. The main disadvantages are (1) if the overall effect of the Mediterranean diet on depression is mostly due to a specific food group, then this effect would be diluted, and (2) although participants may have the same MDS, it does not necessarily mean that the combination and amounts of food groups consumed are the same. Thus, we do not know whether the association between the MDS and depression arises from all components or if it is driven by one or few key food groups within this score. Previous studies focusing on single food groups have provided some evidence that high fish [11], fruit and vegetables [12] and fibre intake [13] in isolation are associated with lower depressive symptoms. However, analysing individual food groups in isolation has limitations as the role of these individual components is investigated without considering the complexity of a whole diet pattern [14]. Consumption of certain food groups are often correlated [15] (e.g., fruit often with vegetables, or fat with sugar). Thus, it would be interesting to know which component(s) of the diet, if any, has the largest association with depression/anxiety both individually and in combination with other dietary components.

To our knowledge four previous mental health focused papers have examined multiple food groups both independently and in combination [16–19]. Results from these papers showed that vegetables, fruit, high fibre, meat, fish, low fat dairy, elevated polyunsaturated fat/saturated fat ratios and low trans-fat were negatively associated with depression, and sugar-sweetened beverages, fast food, snacks and sweets were positively associated with depressive symptoms. All four studies found that, after correction for other food groups, fruit and vegetables remained independently associated with depressive symptoms. Other food groups that were also found to be independently associated with depression were trans-fat intake (in women) [16], snacks/sweets/cookies/fast food [17], meat intake (in women) [18], and low fat dairy and non-refined grains [19]. Thus, there appears to be fairly consistent evidence that low intakes of fruit and vegetables are associated with depressive symptoms, although, the evidence for other food groups is inconsistent. These present studies are limited in their inability to assess clinically diagnosed depression, their restricted populations (university students/white collar civil servants) and their neglect of depression’s comorbidity with anxiety.

The current study analyses a clinical population, which includes persons with current and remitted depression as well as those with anxiety, has several benefits. Firstly, classifying persons as depressed using self-report symptomatology scales can lead to misclassification of depression status due to the overlapping nature of some of symptoms with somatic illnesses. For example, one meta-analysis examining diet quality and depression only included 9 out of 29 studies using standardized diagnostic interviews to ascertain DSM diagnoses [5]. Secondly, anxiety disorders, which are highly comorbid with depression, are also related to dietary intake [20], have been much less examined. Finally, in comparison to current depression, having a history of depression has been associated with healthier dietary intake [21]. It would, therefore, be useful to know whether dietary patterns of those with a history of depression differ from that of healthy controls. Although the relationship between a healthy diet and a lower severity of depressive symptoms has previously been established it would be advantageous to establish whether it is the dietary quality as a whole that is important, or whether the relationship is driven by certain components. As dietary intake is a modifiable risk factor, a more detailed understanding of the relationship between dietary intake and depression and anxiety may provide an extra tool with which clinicians can prevent or treat patients with depression and anxiety disorders.

We have previously shown that poorer diet quality as operationalized by the Mediterranean Diet Score was associated with depressive and anxiety disorders [20]. The aim of the current study, therefore, is to examine the association between the individual food groups which make up the Mediterranean diet with depressive and anxiety (symptom severity and diagnosis) in adults. These food groups will be examined in isolation and in combination with each other to establish which dietary components are independently related to depression and/or anxiety diagnoses and symptom severity.

## Methods

### Source population

The data was sourced from the Netherlands Study of Depression and Anxiety (NESDA) an ongoing longitudinal cohort study designed investigating the course trajectories and consequences of depressive and anxious subjects. The baseline sample consists of 2981 patients [of which 2329 (78%) with a lifetime depressive or anxiety disorder] aged 18–65 years of whom 1979 (66.4%) were female. Patients were recruited in three different Dutch regions from the general population, in general practice and in mental health organisations. General exclusion criteria were an inability to speak Dutch and a primary diagnosis of psychotic, obsessive compulsive, bipolar or severe addiction disorder.

In-depth 4-h interviews in which mental health status, anthropometric measurements, biological measurements and lifestyle factors were assessed at baseline and follow-up which occurred at 2-, 4-, 5- and 9-year intervals. The 9-year assessment included a food frequency questionnaire (FFQ). All participating patients completed written informed consent forms and the research protocol was approved by the Ethical Committee of the participating university. Further details of the NESDA study can be found elsewhere [22].

### Study population

The 9-year follow-up assessment was conducted in 2069 persons. For the present study we included those participants who completed the FFQ (*n* = 1671). Of these, 37 participants were excluded due to improbable energy intake (females: < 500 kcal, > 3500 kcal and males: < 800 kcal, > 4000 kcal [23]) leaving a total sample of 1634. Of the 9-year follow-up participants, those excluded from the current analyses were more likely to be male, younger, less educated and have a higher severity of depression but not anxiety.

### Depressive and anxiety disorder

At each assessment the presence of a DSM-IV depressive [major depressive disorder (MDD), dysthymia] or anxiety disorder (social phobia, agoraphobia, general anxiety disorder and panic) was established using the Composite International Diagnostic Interview (CIDI) version 2.1 [24]. At the 9-year follow-up assessment participants were classified as controls (no lifetime history of depressive or anxiety disorder), current disorder (6-month recency of depressive or anxiety disorders), or remitted disorder (lifetime diagnosis of depressive or anxiety disorder but no current disorder).

Additionally, the severity of symptoms was measured. Depressive symptoms were measured with the 30-item Inventory of Depressive Symptomatology—Self Report (IDS-SR, range 0–84) [25]. The severity of anxiety arousal symptoms was measured using the 21-item Beck Anxiety Inventory (BAI, range 0–63) [26] and the severity of agoraphobia and social phobia with the 15-item Fear Questionnaire (score range 0–120) [27].

### Dietary assessment

Dietary intake was assessed with a 238-item, semi-quantitative FFQ which was based on a validated ethnic Dutch FFQ [28]. The FFQ asked about the frequency, amount and type of food eaten in the past month. Using the Dutch Food Composition Table 2014 [29], daily intakes (g/day) of the 238 food items were calculated. Population medians were imported for missing amounts. Likewise, missing product sort (e.g., full-fat, semi-skimmed or skimmed milk) was replaced with distributions reflecting the population median. The total number of missing items was 1929 (0.6%). The FFQ also included the option to add additional food items consumed within the last week that were not included in the questionnaire. These items were manually re-categorised to comparable food items where possible. Each manual adjustment was made by consensus of two nutritional scientists.

The following 11 food groups (in g/day) were made based on the food groups from the Mediterranean diet score [30]: fruit, vegetables, non-refined grains, legumes, fish, potatoes, olive oil (positively scored), high fat dairy, red and processed meat, poultry (negatively scored). Furthermore, because within the MDS moderate alcohol consumption receives the optimum score and extreme consumptions receive a score of 0, we treated alcohol consumption as a categorical variable. Three categories were non-drinkers (< 36 g ethanol/day), moderate drinkers (≥ 36, < 82 g ethanol/day = reference) and heavy drinkers (≥ 82 g ethanol/day). The overall MDS score was also calculated.

### Other variables

Covariates were selected a priori based on findings from other studies. Gender, age, years of education, partner status (married/living together, single/separated/divorced), smoking status (current, never, former) and physical activity were included as potentially confounding variables. Physical activity during the past week was measured at the 9-year follow-up with the International Physical Activity Questionnaire (IPAQ) and expressed as 1000 MET min/week [31, 32]. Missing values for physical activity (*n* = 124, 7.5%) were imputed using multiple imputation. Five imputations were made and pooled results of the five separate analysis were used.

Antidepressant used in the previous month were asked during interview and classified according to the Anatomical Therapeutic Chemical (ATC) classification. Use of antidepressants was considered when taken at least 50% of the time.

### Statistical analysis

The analyses were conducted using SPSS 22 (IBM Corp., Armonk, NY, USA). Statistical significance was set at *p* < 0.05. Socio-demographic characteristics were described using frequencies and means (medians for non-normally distributed variable). Distributions of the 11 food groups, the MDS and total energy intake were also described.

Separate linear regression models were used to estimate the association of energy intake, the MDS and each of the 11 food groups (continuous g/day and alcohol categories), with depression, anxiety arousal and fear severity (continuous standardised IDS, BAI and FEAR, respectively). To mitigate the effect of differential total intakes due to differing energy needs, which varies according to body size, metabolic efficiency, and physical activity, MDS and food groups were adjusted for energy intake using the energy adjustment method [33]. Thus, residuals were calculated for MDS and the 11 food groups by regressing the MDS/food group as dependent variables against total energy intake (kcal/day) as the independent variable. The utilisation of residuals can be conceptualised as the substitution of that particular food group for a similar number of calories from another food source [23]. The residuals from the linear regression analysis were subsequently standardised to enable comparability among food groups, and used in the analyses. As dietary intake and depression are known to be influenced by partner status, the level of education, and other lifestyle factors, we tested two statistical models. The basic model, which included adjustment for age, gender, and years of education, estimates the fundamental relationship between food groups and depression/anxiety accounting for non-modifiable or relatively stable social demographic factors. The fully adjusted model (i.e., age, sex, education, partner status, smoking status, and physical activity) was additionally adjusted for modifiable characteristics. The associations between having current depression or anxiety, or remitted depression or anxiety compared to controls was analysed using multinomial logistic regression analyses. Again, both basic and fully adjusted models were tested. To enable easier interpretation of the magnitude of the relationships between food groups and depression/anxiety, effect sizes were calculated in fully adjusted models using Pearson’s correlations coefficients for linear models and Cohen’s *d*, defined as the difference in the means between current or remitted diagnosis and controls, divided by the pooled standard deviation of these groups.

To assess the independent effect of any given food groups, a multivariable regression analysis entering all 11 food groups into one fully adjusted model was also performed. Likely, the consumption of certain food groups are correlated with other food groups. Hence, we first examined the correlation (Spearman rho) between food groups and levels of collinearity [variance inflation factor (VIF) and tolerance]. The largest correlation was observed between fruit and vegetables (Spearman rho = 0.34). As the average VIF’s were not substantially above 1, and the maximum VIF was not greater than 10 (max VIF = 1.34) [34, 35] and tolerance levels were not below 0.2 (lowest tolerance was 0.746) we considered multicollinearity not to be a problem. Correction for multiple testing was done for all models using the modified False Discovery rate (Benjamini and Hochberg 1995) method [36].

To negate the potential effect that antidepressant use may have on food intake, a sensitivity analysis was performed excluding persons taking antidepressants known to affect appetite, namely tricyclic antidepressants (TCA) and mirtazapine [37].

Finally, the effect modification of the association between the food groups, MDS score and energy intake by sex was examined. However, as no significant interactions (*p*’s > 0.10) were found the models were not stratified by sex.

## Results

Of the 1634 participants, 414 (25.3%) were diagnosed with a current anxiety or depressive disorder, 886 (52.4%) with a remitted disorder and 334 (20.4%) had no lifetime history of anxiety and depressive disorders. Females made up 67.8% of the participants and the average age was 52.0 years (SD 13.2) (Table 1). The average daily energy intake was 2143 kcal [Standard deviation (SD) 603] and participants scored a mean of 32.7 (SD 4.9) on the MDS.

**Table 1:** Descriptive characteristics and dietary intake of NESDA participants

Characteristic	Total population *n* = 1634	
Age (mean, SD)	52.0	(13.2)
Female (*n*, %)	1108	(67.8)
Education (years), (mean, SD)	13.1	(3.3)
Smoking status (*n*, %)		
Never	550	(33.7)
Current	380	(23.3)
Former	704	(43.1)
Partner Status (*n*, %)		
Single/divorced/separated/widowed	814	(49.8)
Married/Living together	820	(50.2)
Physical activity 1000 MET mins/week (mean, SD)	3.8	(3.2)
IDS depression score (median, IQR)	11.0	(6.0–21.0)
BAI anxiety score (median, IQR)	5.0	(1.0–11.0)
FEAR phobia score (median, IQR)	10.0	(3.0–23.0)
Disorder status (*n*, %)		
Control	334	(20.4)
Remitted depression/anxiety	886	(54.2)
Current depression/anxiety	414	(25.3)
Energy intake (kcal) (mean, SD)	2143.6	(602.9)
Total MDS score	32.7	(4.9)
Non-refined grains, g/day (median, IQR)	127.5	(85.3–178.5)
Vegetables, g/day (median, IQR)	158.9	(105.1–223.6)
Fruit, g/day (median, IQR)	163.9	(75.5–253.8)
Fish, g/day (median, IQR)	16.1	(7.7–30.0)
Olive Oil, g/day (median, IQR)	3.7	(0.1–7.3)
Red and processed meat, g/day (median, IQR)	54.0	(30.1–83.9)
Potatoes, g/day (median, IQR)	51.1	(25.1–86.4)
Legumes and soya, g/day (median, IQR)	24.3	(11.0–46.4)
High fat dairy, g/day (median, IQR)	71.1	(30.5–134.8)
Poultry, g/day (median, IQR)	11.4	(7.1–25.1)
Alcohol consumption (*n*, %)		
Non-drinker	331	(20.3)
Heavy drinker	14	(0.9)
Moderate drinker	1289	(78.9)

*SD* standard deviation, *IQR* inter quartile range, *IDS* Inventory of Depression Symptomology, *BAI* Beck Anxiety Inventory, *MDS* Mediterranean diet score

Table 2 presents the association of total energy intake, MDS and food group intake residuals with the severity of depression and anxiety symptoms. After adjustment for age, sex and education and taking multiple testing into account, higher MDS score, and higher consumption of non-refined grain, vegetables, and fruit (for FEAR score only) were significantly associated with lower standardised IDS, BAI and FEAR scores. Non-drinking (compared to moderate drinking) was also significantly associated with higher IDS and BAI. Higher energy intake was significantly associated with BAI. Of all food characteristics, the overall MDS score had the strongest association [IDS = standardised beta (*β*) − 0.13 95% confidence intervals (95% CI) − 0.18, − 0.08 and BA I = *β* − 0.11 95% CI − 0.16, − 0.06, FEAR = *β* − 0.08 95% CI − 0.13, − 0.03). The comparative effect sizes (standardised beta-coefficients) were IDS: *r* − 0.11, 95% CI − 0.17, − 0.06, BAI *r* − 0.09 95% CI − 0.14, − 0.04 FEAR: *r* 0.06 95% CI 0.01, 0.12 (Supplementary Table 1). Of the individual food groups, non-refined grains intake (IDS: *β* − 0.10 *r* − 0.10, 95% CI − 0.15, − 0.05, BAI: *β* − 0.07 *r* − 0.06, 95% CI − 0.12, − 0.02), vegetables intake (FEAR: *β* − 0.11 *r* − 0.01, 95% CI − 0.16, − 0.06) and being a non-drinker (vs. Moderate drinker IDS: *β* 0.09 *r* 0.10, 95% CI 0.04, 0.14, BAI: *β* 0.08 *r* 0.08, 95% CI 0.03, 0.13) showed the strongest associations. Post-hoc analysis, using the residual of only the vegetable and grain components of the MDS score, showed that the *β*-coefficients were larger than that of the total MDS score for depressive and anxiety symptoms (*β*-coefficients of − 1.43, − 1.14 and − 0.10, respectively for IDS, BAI and FEAR versus − 1.28, − 1.07 and − 0.09).The *β*-coefficients for clinical diagnosis were the same for both the total score and partial score. Additional adjustment for modifiable lifestyle factors, namely partner status, physical activity, and smoking status did not change these results substantially (Table 2 and Supplementary Fig. 1).

**Table 2:** Separate association between standardized food group residuals, energy and MDS with the standardised severity of depression (IDS), anxiety (BAI) and phobias (FEAR)

	IDS Score (*n* = 1616)						BAI (*n* = 1612)						FEAR (*n* = 1613)					
	Model 1			Model 2			Model 1			Model 2			Model 1			Model 2		
	*β*	95% CI	*p* value	*β*	95% CI	*p* value	*β*	95% CI	*p* value	*β*	95% CI	*p* value	*β*	95% CI	*p* value	*β*	95% CI	*p* value
Energy (kcal/day)	0.05	(0.00, 1.10)	0.07	0.06	(0.00, 1.11)	0.02	**0.07**	**(0.02, 0.12)**	**0.01**	**0.08**	**(0.03, 0.13)**	< **0.01**	0.06	(0.01, 1.10)	0.03	**0.07**	**(0.02, 1.12)**	**0.01**
MDS Score	**− 0.13**	**(− 0.18, − 0.08)**	< **0.01**	**− 0.12**	**(− 0.17, − 0.07)**	< **0.01**	**− 0.11**	**(− 0.16, − 0.06)**	< **0.01**	**− 0.09**	**(− 0.14, − 0.04)**	< **0.01**	**− 0.08**	**(− 0.13, − 0.03)**	< **0.01**	**− 0.08**	**(− 0.13, − 0.03)**	< **0.01**
Food group residuals																		
Non-refined grains	**− 0.10**	**(− 0.15, − 0.05)**	< **0.01**	**− 0.09**	**(− 0.14, − 0.05)**	< **0.01**	**− 0.07**	**(− 0.12, − 0.02)**	< **0.01**	**− 0.06**	**(− 0.11, − 0.01)**	**0.01**	**−** 0.02	(**−** 0.07, **−** 0.03)	0.52	**−** 0.01	(**−** 0.06, **−** 0.03)	0.65
Vegetables	**− 0.07**	**(− 0.12, − 0.02)**	**0.01**	**−** 0.06	(**−** 0.11, **−** 0.01)	0.03	**− 0.06**	**(− 0.11, − 0.01)**	**0.01**	**−** 0.05	(**−** 0.10, **−** 0.00)	0.04	**− 0.11**	**(− 0.16, − 0.06)**	< **0.01**	**− 0.10**	**(− 0.15, − 0.05)**	< **0.01**
Fruit	**−** 0.05	(**−** 0.10, 0.00)	0.04	**−** 0.04	(**−** 0.09, 0.01)	0.14	**−** 0.02	(**−** 0.07, 0.03)	0.48	0.00	(**−** 0.05, 0.05)	0.96	**− 0.09**	**(− 0.14, 0.04)**	< **0.01**	**− 0.08**	**(− 0.13, 0.03)**	< **0.01**
Fish	**−** 0.03	(**−** 0.07, 0.02)	0.30	**−** 0.02	(**−** 0.07, 0.03)	0.35	**−** 0.04	(**−** 0.08, 0.01)	0.15	**−** 0.04	(**−** 0.08, 0.01)	0.16	**−** 0.05	(**−** 0.10, 0.00)	0.05	**−** 0.05	(**−** 0.09, 0.00)	0.06
Olive oil	**−** 0.02	(**−** 0.07, 0.03)	0.34	**−** 0.02	(**−** 0.07, 0.03)	0.37	**−** 0.02	(**−** 0.07, 0.03)	0.39	**−** 0.02	(**−** 0.07, 0.03)	0.42	**−** 0.02	(**−** 0.07, 0.03)	0.39	**−** 0.02	(**−** 0.07, 0.03)	0.39
Red and processed meat^a^	**−** 0.02	(**−** 0.07, 0.03)	0.42	**−** 0.02	(**−** 0.07, 0.03)	0.36	**−** 0.02	(**−** 0.07, 0.03)	0.46	**−** 0.03	(**−** 0.07, 0.02)	0.31	0.00	(**−** 0.05, 0.05)	0.97	0.00	(**−** 0.05, 0.05)	0.88
Potatoes	**−** 0.02	(**−** 0.07, 0.03)	0.47	**−** 0.02	(**−** 0.07, 0.03)	0.50	**−** 0.05	(**−** 0.10, 0.00)	0.06	**−** 0.05	(**−** 0.10, 0.00)	0.06	0.02	(**−** 0.03, 0.07)	0.34	0.02	(**−** 0.03, 0.07)	0.38
Legumes and soya	**−** 0.01	(**−** 0.06, 0.04)	0.63	**−** 0.02	(**−** 0.06, 0.03)	0.50	0.00	(**−** 0.05, 0.05)	0.97	0.00	(**−** 0.05, 0.05)	0.97	0.03	(**−** 0.02, 0.08)	0.18	0.03	(**−** 0.02, 0.08)	0.20
High fat dairy^a^	0.02	(**−** 0.03, 0.06)	0.51	0.01	(**−** 0.04, 0.06)	0.65	0.02	(**−** 0.02, 0.07)	0.31	0.02	(**−** 0.03, 0.07)	0.41	0.00	(**−** 0.04, 0.05)	0.86	0.00	(**−** 0.04, 0.05)	0.90
Poultry^a^	0.03	(**−** 0.01, 0.08)	0.17	0.04	(**−** 0.01, 0.08)	0.14	0.01	(**−** 0.04, 0.06)	0.68	0.01	(**−** 0.04, 0.06)	0.66	0.01	(**−** 0.04, 0.06)	0.72	0.01	(**−** 0.04, 0.06)	0.66
Heavy drinker^a^	**−** 0.01	(**−** 0.06, 0.03)	0.54	**−** 0.02	(**−** 0.07, 0.03)	0.43	0.01	(**−** 0.04, 0.06)	0.68	0.00	(**−** 0.04, 0.05)	0.87	0.00	(**−** 0.05, 0.05)	0.99	0.00	(**−** 0.05, 0.05)	0.92
Non-Drinker^a^	**0.09**	**(0.04, 0.14)**	< **0.01**	**0.09**	**(0.05, 0.14)**	< **0.01**	**0.08**	**(0.03, 0.13)**	< **0.01**	**0.08**	**(0.03, 0.13)**	< **0.01**	0.06	(0.01, 0.11)	0.03	0.06	(0.01, 0.11)	0.02

Model 1: age, sex, education (years)

Model 2: Model 1 + partner status, physical activity, smoking status

Bold: significant after correction for multiple testing

^a^In the MDS these items are negatively scored, meaning that the direction of association is expected to be the opposite (*b* < 0) of the other food groups

A higher MDS score was significantly associated with lower odds of having a current disorder compared to controls after adjustment for multiple testing in the basic model [odds ratio (OR) 0.77, Cohen’s *d* − 0.07 95% CI 0.66, 0.90] (Table 3and supplementary Fig. 2). Although higher energy intake was associated with having a current disorder, it did not reach statistical significance after allowance for multiple testing. Of the individual food groups, a higher intake of non-refined grains was significantly associated with lower odds of a current disorder, and being a non-drinker had significantly higher odds of having a current disorder compared to moderate drinking. Again, the odds ratios only changed marginally after additional adjustment for lifestyle factors (Table 3). Those with a remitted disorder did not differ significantly in food intake from controls.

**Table 3:** Association between standardized food group residuals, energy intake and MDS with current depression/anxiety and remitted depression/anxiety compared to controls (*n* = 1634)

	Remitted anxiety/depression compared to controls						Current anxiety/depression compared to controls					
	Model 1			Model 2			Model 1			Model 2		
	Odds ratio	95% CI	*p* value	Odds ratio	95% CI	*p* value	Odds ratio	95% CI	*p* value	Odds ratio	95% CI	*p* value
Energy (kcal/day)	1.08	(0.94–1.24)	0.26	1.07	(0.93–1.23)	0.36	1.17	(1.00–1.37)	0.05	1.19	(1.01–1.39)	0.04
MDS Score	0.96	(0.84–1.10)	0.54	0.98	(0.85–1.12)	0.75	0.77	**(0.66–0.90)**	< **0.01**	**0.80**	**(0.68–0.93)**	< **0.01**
Food group residuals												
Non-refined grains	0.88	(0.78–1.00)	0.05	0.92	(0.81–1.04)	0.19	**0.83**	**(0.72–0.96)**	**0.01**	0.86	(0.74–1.00)	0.05
Vegetables	1.05	(0.92–1.20)	0.46	1.05	(0.92–1.20)	0.46	0.86	(0.74–1.01)	0.06	0.88	(0.75–1.03)	0.11
Fruit	0.95	(0.84–1.08)	0.48	0.98	(0.86–1.12)	0.79	0.84	(0.72–0.98)	0.03	0.88	(0.75–1.02)	0.10
Fish	0.99	(0.87–1.12)	0.85	0.97	(0.86–1.11)	0.69	0.97	(0.84–1.13)	0.70	0.97	(0.83–1.12)	0.67
Olive oil	1.09	(0.95–1.24)	0.22	1.08	(0.94–1.23)	0.29	0.99	(0.85–1.16)	0.91	0.99	(0.84–1.15)	0.87
Red and processed meat^a^	0.98	(0.86–1.11)	0.70	0.96	(0.85–1.10)	0.59	0.92	(0.79–1.07)	0.26	0.91	(0.78–1.05)	0.20
Potatoes	0.97	(0.85–1.11)	0.66	0.99	(0.87–1.13)	0.90	0.92	(0.79–1.07)	0.29	0.94	(0.80–1.09)	0.39
Legumes and soya	1.04	(0.91–1.18)	0.58	1.04	(0.91–1.19)	0.55	1.04	(0.90–1.21)	0.58	1.04	(0.89–1.20)	0.63
High fat dairy^a^	0.95	(0.84–1.08)	0.46	0.95	(0.84–1.08)	0.46	1.00	(0.87–1.16)	0.98	0.99	(0.86–1.15)	0.92
Poultry^a^	1.05	(0.92–1.20)	0.47	1.05	(0.92–1.21)	0.44	1.10	(0.95–1.27)	0.22	1.11	(0.95–1.29)	0.19
Heavy drinker	0.94	(0.84–1.05)	0.30	0.93	(0.83–1.04)	0.19	0.96	(0.83–1.10)	0.53	0.94	(0.82–1.08)	0.38
Non-drinker	1.03	(0.89–1.19)	0.68	1.07	(0.93–1.23)	0.36	**1.22**	**(1.04–1.42)**	**0.01**	**1.25**	**(1.07–1.46)**	< **0.01**

Model 1: age, sex, education (years)

Model 2: Model 1 + partner status, physical activity, smoking status

Bold: significant after correction for multiple testing

^a^In the MDS these items are negatively scored, meaning that the direction of association is expected to be the opposite (OR < 1) of the other food groups

Combining all the food groups into one (fully adjusted) model showed a similar pattern to the analysis of individual food groups. Thus, higher consumption of non-refined grains was associated with lower IDS and BAI severity scores and being a non-drinker was associated with higher scores, whilst higher vegetable consumption was associate with lower FEAR score (Table 4). Both higher non-refined grain consumption and being a non-drinker remained significantly associated with having a current disorder after correction for multiple testing (Table 5).

**Table 4:** The association between standardized food group residuals with the standardized severity of depression (IDS), anxiety (BAI) and FEAR cor**r**ected for all other food groups (*n* = 1634)

Food group residuals	IDS Score (*n* = 1616)^a^			BAI (*n* = 1612)^a^			FEAR (*n* = 1613)^a^		
	*β*	95% CI	*p* value	*β*	95% CI	*p* value	*β*	95% CI	*p* value
Non-refined grains	**− 0.11**	**(− 0.16, − 0.06)**	**0.00**	**− 0.07**	**(− 0.12, − 0.02)**	**0.01**	**−** 0.02	(**−** 0.07, 0.03)	0.37
Vegetables	**−** 0.04	(**−** 0.10, **−** 0.01)	0.12	**−** 0.05	(**−** 0.10, **−** 0.01)	0.10	**− 0.10**	**(− 0.15, − 0.04)**	**0.00**
Fruit	**−** 0.03	(**−** 0.08, **−** 0.02)	0.20	0.01	(**−** 0.04, 0.06)	0.79	**−** 0.06	(**−** 0.11, **−** 0.00)	0.03
Fish	**−** 0.01	(**−** 0.06, 0.04)	0.60	**−** 0.03	(**−** 0.08, 0.02)	0.23	**−** 0.02	(**−** 0.07, 0.03)	0.53
Olive oil	**−** 0.01	(**−** 0.06, 0.04)	0.68	0.00	(**−** 0.06, 0.05)	0.86	0.00	(**−** 0.05, 0.05)	0.89
Red and processed meat^b^	**−** 0.05	(**−** 0.11, **−** 0.00)	0.05	**−** 0.03	(**−** 0.08, 0.03)	0.30	**−** 0.02	(**−** 0.07, 0.04)	0.59
Potatoes	**−** 0.01	(**−** 0.06, 0.04)	0.57	**−** 0.05	(**−** 0.10, 0.00)	0.06	0.02	(**−** 0.04, 0.07)	0.55
Legumes and soya	**−** 0.01	(**−** 0.06, 0.04)	0.63	0.01	(**−** 0.04, 0.06)	0.80	0.06	(0.01, 0.11)	0.03
High fat dairy^b^	**−** 0.02	(**−** 0.07, 0.03)	0.48	0.00	(**−** 0.05, 0.05)	0.99	**−** 0.01	(**−** 0.06, 0.04)	0.61
Poultry^b^	0.05	(0.00, 0.10)	0.08	0.02	(**−** 0.03, 0.07)	0.43	0.02	(**−** 0.03, 0.07)	0.42
Heavy drinker	**−** 0.04	(**−** 0.09, 0.01)	0.13	**−** 0.01	(**−** 0.06, 0.04)	**−** 0.71	**−** 0.01	(**−** 0.06, 0.03)	0.44
Non-drinker	**0.09**	**(0.04, 0.14)**	**0.00**	**0.08**	**(0.05, 0.10)**	**0.00**	0.05	(0.00, 0.10)	0.05

Bold: significant after correction for multiple testing

^a^Adjusted for age, sex, education (years), partner status physical activity, smoking status

^b^In the MDS these items are negatively scored, meaning that the direction of association is expected to be the opposite (*b* < 0) of the other food groups

**Table 5:** The association between standardized food group residuals with current depression/anxiety and remitted depression/anxiety compared to controls corrected for all other food groups (*n* = 1634)

Food group residuals	Remitted depression/anxiety compared to controls			Current depression/anxiety compared to controls		
	Model 1^a^			Model 1^a^		
	Odds ratio	95% CI	*p* value	Odds ratio	95% CI	*p* value
Non-refined grains	0.89	(0.78, 1.01)	0.08	**0.82**	**(0.71, 0.96)**	**0.01**
Vegetables	1.04	(0.89, 1.21)	0.63	0.89	(0.74, 1.06)	0.20
Fruit	0.95	(0.83, 1.10)	0.51	0.87	(0.74, 1.03)	0.10
Fish	0.95	(0.83, 1.09)	0.49	0.99	(0.85, 1.16)	0.94
Olive oil	1.07	(0.93, 1.23)	0.36	1.02	(0.86, 1.20)	0.83
Red and processed meat^b^	0.92	(0.80, 1.07)	0.29	0.84	(0.70, 1.00)	0.05
Potatoes	0.99	(0.86, 1.14)	0.89	0.94	(0.80, 1.11)	0.47
Legumes and soya	1.02	(0.88, 1.17)	0.83	1.04	(0.89, 1.22)	0.65
High fat dairy^b^	0.93	(0.82, 1.06)	0.30	0.93	(0.86, 1.01)	0.36
Poultry^b^	1.06	(0.92, 1.23)	0.43	1.16	(0.98, 1.36)	0.09
Heavy drinker	0.91	(0.81, 1.02)	0.12	0.90	(0.84, 0.97)	0.14
Non-Drinker	1.08	(0.93, 1.25)	0.30	**1.26**	**(1.08, 1.46)**	**0.00**

Bold: significant after correction for multiple testing

^a^Adjusted for age, sex, education (years), partner status physical activity, smoking status

^b^In the MDS these items are negatively scored, meaning that the direction of association is expected to be the opposite (OR < 1) of the other food groups

Excluding participants using antidepressant drugs affecting appetite [i.e., TCA’s or mirtazapine (*n* excluded = 141)] did not alter the association between food groups and the severity of depression/anxiety (data not shown).

## Discussion

Examining food groups in isolation showed that higher vegetable intake was related to lower depression, anxiety and fear severity. Higher non-refined grain consumption was significantly related to lower depression and anxiety arousal severity and lower odds of having a current clinically diagnosed disorder compared to controls and these relationships persisted after adjustment for other food groups. Additionally compared to being a moderate drinker, being a non-drinker was associated with greater depression and anxiety severity and greater odds of being currently depressed Thus, these elements appear to be the most important factors within the Mediterranean diet. Analysing the diet as a whole using the MDS showed that a less healthy diet was significantly associated with both depression/anxiety diagnosis and increased symptom severity. Total energy intake was associated with a higher severity of anxiety symptoms, depressive symptoms and a diagnosis of depression/anxiety, although the latter two findings were not statistically significant when allowance was made for multiple testing. In general effect sizes were small, implying that despite significant relationships between diet and depression/anxiety, the impact of food groups on depression was small for the individual patient, but may be of clinical importance on the population level.

Generally, the direction of association of individual food groups was in line with expectations. Thus, for both outcomes, higher consumption of non-refined grains, vegetables, fruit, potatoes, fish and olive oil were inversely related to depression or anxiety severity or lower odds of a current diagnosis, whilst higher consumption of poultry and high fat dairy products was positively associated with higher depressive/anxiety symptoms and depression/anxiety disorder. Only consumption of red and processed meat was not consistent with expectations as a higher intake tended towards lower severity score/odds of a current disorder. This and the fact that the MDS score had the strongest associations, suggests that overall it is the cumulative, and potentially synergistic effect of nutrients from different food groups that are linked to mental health.

In line with previous studies, we found that the Mediterranean diet was inversely associated with depression [5, 6, 38]. Our participants have slightly lower MDS (mean 32.7 SD 4.9) compared to traditional diets of people living in the Mediterranean area according to the MEDIS study (mean 33 SD 4.0), and where according to the Ikaria study, the healthiest populations score an average of 38.0 (SD 2.7 men, 3.0 women) [38].

Higher energy intake was associated with a higher severity of anxiety symptoms as well as having a tendency to be associated with current depression/anxiety disorder. This is not surprising as previous studies found that healthier diets, the components of which tend to have lower energy densities, were associated with less depressive symptoms [5]. Furthermore, higher BMI, which may result from excess energy intake, has been associated with depression [39].

Studies examining individual food groups had mixed findings, partly due to the varying combination of food groups examined. However, in accordance with our study, higher vegetable consumption has been consistently associated with less depression in studies that investigated multiple food groups simultaneously [16–19, 40] and vegetables as single food group [5, 12]. Similar to our study, two studies found that higher non-refined grain consumption was associated with a lower incidence of depression [19, 40]. Additionally, two other studies also found that increased fibre intake was associated with lower depression [13, 16]. Interestingly, the observation that the direction of associations between red and processed meat consumption was not consistent with our expectations (i.e., we found higher consumption tending towards a lower odds of depression/anxiety disorder) has been reported elsewhere in females [18]. The authors of this study suggested that possibly meat consumption was a reflection of a better mood state, rather than that a higher meat consumption adversely affected mood. Other evidence for associations of meat consumption with depression comes from studies investigating Western dietary pattern as a whole [41], rather than its single components (i.e., high intake of red and processed meat, refined grains, sweets and high fat dairy products). Our finding of a lack of association with high fat dairy products and red/processed meat suggests that perhaps the other elements of the Western diet, namely high sugar and fat consumption, drive the association between a Western dietary pattern and depressive symptom, which has been confirmed by other studies [42, 43].

Contrary to other studies [11], we found no associations between fish and depressive symptoms. A meta-analysis of 26 studies involving 150,278 participants indicated that high-fish consumption reduced the risk of depressive symptoms. Lack of findings could be due to the generally low levels of fish consumption in the Netherlands, one study reported reduced risk was achieved at 50 g/day [44], or an unfavourable ratio of white fish to oily fish (high in omega 3-fatty acids). The average daily fish intake in the Netherlands was only 53 g/week of which 25% was fatty fish [45].

This is the first study to analyse individual food group consumption and its association with anxiety symptom severity. Those with increased anxiety severity have similar food group consumption patterns to those with increased depressive symptoms, also having lower intakes of non-refined grains and vegetables. Similarly, increased symptoms of agoraphobia and social phobias, as measured by the FEAR questionnaire, were also significantly associated with lower vegetable intake along with lower fruit intake.

The MDS classifies both high alcohol and non-alcohol consumption as unhealthy. We found that compared to moderate alcohol intake, being a non-drinker was significantly associated with higher odds of having a current depression and/or anxiety disorder and significantly associated with higher depression and anxiety symptom severity. This might be explained by the fact that depressed or anxious persons are often advised to minimalize the intake of alcohol to improve mood or because some its use may interact with antidepressant use. We cannot exclude the possibility that the association between low alcohol consumption and depression is observed due to reverse causality. Unexpectedly, heavy drinking was not associated with increased odds of a disorder/increased disorder severity. This could be due to insufficient statistical power as there were only 14 heavy drinkers. Furthermore, previous literature has shown that depression is related to drinking larger quantities per occasion as opposed to the frequency of drinking [46]. Indeed, hazardous and harmful alcohol use has been associated with depression and anxiety in this cohort [47].

Mechanisms underlying the association between the dietary quality and depression/anxiety are complex and arguments can be made for bidirectional relationships. First, poor (or increased) appetite, weight loss (or gain), poor motivation, and low energy levels are symptoms typically found in depressed persons [48]. This often leads to changes in energy intake and a reduction in personal health behaviours [49], and given that healthy diets typically require more time and cooking skills [50], whereas unhealthy foods are quick and easy to prepare, it could be expected that the diet quality may become compromised. Second, deficiencies in certain vitamins [51], minerals [52], and essential fatty acids (such as long chain n-3 polyunsaturated fatty acids derived from fatty fish) [53] may impact depression by directly influencing biological pathways associated with the pathophysiology of depression. Low levels of folic acid, which is abundant in non-refined grains and vegetables, and zinc, a mineral found in non-refined grain products, have both been associated with depression [54, 55]. Vegetables are an important source of minerals, fibre, alpha-linolenic acid (i.e., 18:3n-3 PUFA), and vitamins, and other anti-oxidants. Anti-oxidants counteract free radicals and may, therefore, help alleviate oxidative stress, which has been shown to be increased in depressed persons [56]. Third, diet may influence depression and anxiety indirectly through negatively affecting the gut microbiome and introducing low-grade inflammation, which in turn poses a risk for depression. Alternatively, diet may influence depression and anxiety indirectly through poor metabolic health. Metabolic conditions such as obesity [39], metabolic syndrome [57] and diabetes type 2 [58] have all been associated with depression and consuming an unhealthy diet increases the risk of these metabolic diseases [59, 60]. Finally, we should consider the possibility that the association between diet quality and depression may have been confounded by social economic status (SES) and income. An increased risk of depression is typically associated with lower SES and income [61, 62]. Many of the food groups associated with lower depression and anxiety are typically more expensive and more often consumed by those of higher income, SES and education, and although we adjusted for education level, there may have been residual confounding by SES [63].

The strengths of this study are that we were able to analyse both depression and anxiety disorders, which are highly comorbid, as well as being able to compare symptom severity scores with clinical diagnosis in a population selected to represent a broad range of depression and anxiety stages and severities. Another strength is that the FFQ included frequencies and serving sizes, thereby making the estimation of food intake more accurate. There were, however, also some limitations. The primary limitation is the cross-sectional design, thus precluding any assumptions about the temporal direction. Secondly, assessing dietary intake with a self-report FFQ is prone to misreporting and recall bias. Reporting accuracy in the FFQ could possibly be associated with disorder severity as depression can adversely influence several cognitive functions. Over and underestimation, the latter particularly in obese subjects, of actual food consumption, poor recall and the omission of frequently eaten items from the FFQ are inherent problems. However, we removed those with extreme energy intakes, and added other frequently consumed products.

## Conclusion

We can conclude that non-refined grains and, to some extent, vegetables and alcohol consumption appeared to be the driving variables for the associated the total MDS score and depression/anxiety. However, the combined effect of the whole diet remains important for mental health. These associations were not restricted to depressive symptoms, but also to clinically diagnosed depression and anxiety disorders which had not previously been established. The association was not apparent in those who had recovered from depression or anxiety disorders. It should be explored whether an increase consumption of non-refined grains and vegetables may help to prevent or reduce depression and anxiety.

## Electronic supplementary material

Below is the link to the electronic supplementary material.

Supplementary material 1 (DOCX 93 KB)

Supplementary material 2 (DOCX 15 KB)

## References

[1] Barber RM, Bell B, Global Burden of Disease Study 2013 Collaborators T (2015). Global, regional, and national incidence, prevalence, and years lived with disability for 301 acute and chronic diseases and injuries in 188 countries, 1990–2013: a systematic analysis for the Global Burden of Disease Study 2013. Lancet.

[2] World Health Organization (2017) Fact sheet: depression. http://www.who.int/mediacentre/factsheets/fs369/en/. Accessed 4 Jan 2018

[3] Melartin TK, Rytsälä HJ, Leskelä US (2002). Current comorbidity of psychiatric disorders among DSM-IV major depressive disorder patients in psychiatric care in the Vantaa Depression Study. J Clin Psychiatry.

[4] Baxter AJ, Vos T, Scott KM (2014). The global burden of anxiety disorders in 2010. Psychol Med.

[5] Molendijk M, Molero P, Ortuño Sánchez-Pedreño F (2018). Diet quality and depression risk: a systematic review and dose-response meta-analysis of prospective studies. J Affect Disord.

[6] Lassale C, Batty GD, Baghdadli A (2018). Healthy dietary indices and risk of depressive outcomes: a systematic review and meta-analysis of observational studies. Mol Psychiatry.

[7] Nicolaou M, Vermeulen E, Elstgeest L (2017). A meta-analysis of the role of a priori dietary indices in depression among 7 cohorts; the Moodfood project. Ann Nutr Metab.

[8] Rahe C, Unrath M, Berger K (2014). Dietary patterns and the risk of depression in adults: a systematic review of observational studies. Eur J Nutr.

[9] Panagiotakos D, Kalogeropoulos N, Pitsavos C (2009). Validation of the MedDietScore via the determination of plasma fatty acids. Int J Food Sci Nutr.

[10] Parletta N, Zarnowiecki D, Cho J (2017). A Mediterranean-style dietary intervention supplemented with fish oil improves diet quality and mental health in people with depression: a randomized controlled trial (HELFIMED). Nutr Neurosci.

[11] Li F, Liu X, Zhang D (2016). Fish consumption and risk of depression: a meta-analysis. J Epidemiol Community Health.

[12] Liu X, Yan Y, Li F, Zhang D (2016). Fruit and vegetable consumption and the risk of depression: a meta-analysis. Nutrition.

[13] Miki T, Eguchi M, Kurotani K (2016). Dietary fiber intake and depressive symptoms in Japanese employees: the Furukawa nutrition and health study. Nutrition.

[14] McNaughton SA (2010). Dietary patterns and diet quality: approaches to assessing complex exposures in nutrition. Australas Epidemiol.

[15] Waijers PMCM, Feskens EJM, Ocké MC (2007). A critical review of predefined diet quality scores. Br J Nutr.

[16] Akbaraly TN, Sabia S, Shipley MJ (2013). Adherence to healthy dietary guidelines and future depressive symptoms: evidence for sex differentials in the Whitehall II study. Am J Clin Nutr.

[17] El Ansari W, El Adetunji H, Oskrochi R (2014). Food and mental health: relationship between food and perceived stress and depressive symptoms among university students in the United Kingdom. Cent Eur J Med.

[18] Mikolajczyk RT, El Ansari W, Maxwell AE (2009). Food consumption frequency and perceived stress and depressive symptoms among students in three European countries. Nutr J.

[19] Rius-ottenheim N, Kromhout D, Sijtsma FPC et al (2017) Dietary patterns and mental health after myocardial infarction. PLoS One 1–13 10.1371/journal.pone.0186368 PMC5642887 29036212

[20] Gibson-Smith D, Bot M, Brouwer IA (2018). Diet quality in persons with and without depressive and anxiety disorders. J Psychiatr Res.

[21] Jacka FN, Cherbuin N, Anstey KJ, Butterworth P (2015). Does reverse causality explain the relationship between diet and depression?. J Affect Disord.

[22] Penninx BWJH, Beekman ATF, Smit JH (2008). The Netherlands study of depression and anxiety (NESDA): rationale, objectives and methods. Int J Methods Psychiatr Res.

[23] Willett WC (2013). Nutritional epidemiology.

[24] Wittchen HU (1994). Reliability and validity studies of the WHO—Composite International Diagnostic Interview (CIDI): a critical review. J Psychiatr Res.

[25] Rush AJ, Gullion CM, Basco MR (1996). The inventory of depressive symptomatology (IDS): psychometric properties. Psychol Med.

[26] Beck AT, Epstein N, Brown G, Steer RA (1988). An inventory for measuring clinical anxiety: psychometric properties. J Consult Clin Psychol.

[27] Marks IM, Mathews AM (1979). Brief standard self-rating for phobic patients. Behav Res Ther.

[28] Siebelink E, Geelen A, de Vries JHM (2011). Self-reported energy intake by FFQ compared with actual energy intake to maintain body weight in 516 adults. Br J Nutr.

[29] National Institute for Public Health and the Environment (RIVM) (2011). Dutch Nutrients Database 2011/Version 3.

[30] Panagiotakos DB, Pitsavos C, Arvaniti F, Stefanadis C (2007). Adherence to the Mediterranean food pattern predicts the prevalence of hypertension, hypercholesterolemia, diabetes and obesity, among healthy adults; the accuracy of the MedDietScore. Prev Med (Baltim).

[31] Kurtze N, Rangul V, Hustvedt B-E (2008). Reliability and validity of the international physical activity questionnaire in the Nord–Trøndelag health study (HUNT) population of men. BMC Med Res Methodol.

[32] Ekelund U, Sepp H, Brage S (2006). Criterion-related validity of the last 7-day, short form of the International Physical Activity Questionnaire in Swedish adults. Public Health Nutr.

[33] Willett WC, Howe GR, Kushi L (1997). Adjustment for total energy intake in epidemiologic studies. Am J Clin Nutr.

[34] Bowerman BL, O’Connell RT (1990). Linear statistical models: an applied approach.

[35] Feild A (2012). Discovering statistics using IBM SPSS statistics.

[36] Narum SR (2006). Beyond Bonferroni: less conservative analyses for conservation genetics. Conserv Genet.

[37] Fava M (2000). Weight gain and antidepressants. J Clin Psychiatry.

[38] Panagiotakos DB, Chrysohoou C, Siasos G (2011). Sociodemographic and lifestyle statistics of oldest old people (>80 years) living in Ikaria island: the Ikaria study. Cardiol Res Pract.

[39] Luppino FS, de Wit LM, Bouvy PF (2010). Overweight, obesity, and depression: a systematic review and meta-analysis of longitudinal studies. Arch Gen Psychiatry.

[40] Gangwisch JE, Hale L, Garcia L (2015). High glycemic index diet as a risk factor for depression: analyses from the Women’ s Health Initiative. Am J Clin Nutr.

[41] Li Y, Lv MR, Wei YJ (2017). Dietary patterns and depression risk: a meta-analysis. Psychiatry Res.

[42] Knüppel A, Shipley MJ, Llewellyn CH, Brunner EJ (2017). Sugar intake from sweet food and beverages, common mental disorder and depression: prospective findings from the Whitehall II study. Sci Rep.

[43] Vermeulen E, Stronks K, Snijder MB (2017). A combined high-sugar and high-saturated-fat dietary pattern is associated with more depressive symptoms in a multi-ethnic population: the HELIUS (Healthy Life in an Urban Setting) study. Public Health Nutr.

[44] Grosso G, Micek A, Marventano S (2016). Dietary n-3 PUFA, fish consumption and depression: a systematic review and meta-analysis of observational studies. J Affect Disord.

[45] Hengeveld LM, Praagman J, Beulens JWJ (2018). Fish consumption and risk of stroke, coronary heart disease, and cardiovascular mortality in a Dutch population with low fish intake. Eur J Clin Nutr.

[46] Graham K, Massak A, Demers A, Rehm J (2007). Does the association between alcohol consumption and depression depend on how they are measured?. Alcohol Clin Exp Res.

[47] Boschloo L, Vogelzangs N, Smit JH (2011). Comorbidity and risk indicators for alcohol use disorders among persons with anxiety and/or depressive disorders. J Affect Disord.

[48] American Psychiatric Association (APA) (2013). Diagnostic and statistical manual of mental disorders.

[49] Allgöwer A, Wardle J, Steptoe A (2001). Depressive symptoms, social support, and personal health behaviors in young men and women. Health Psychol.

[50] Jabs J, Devine CM (2006). Time scarcity and food choices: an overview. Appetite.

[51] Bender A, Hagan KE, Kingston N (2017). The association of folate and depression: a meta-analysis. J Psychiatr Res.

[52] Wang J, Um P, Dickerman B, Liu J (2018). Zinc, magnesium, selenium and depression: a review of the evidence, potential mechanisms and implications. Nutrients.

[53] Thesing CS, Bot M, Milaneschi Y (2018). Omega-3 and omega-6 fatty acid levels in depressive and anxiety disorders. Psychoneuroendocrinology.

[54] Gilbody S, Lightfoot T, Sheldon T (2007). Is low folate a risk factor for depression? A meta-analysis and exploration of heterogeneity. J Epidemiol Community Health.

[55] Li L, Song Z, Zhang D (2017). Dietary zinc and iron intake and risk of depression: a meta-analysis. Psychiatry Res.

[56] Black CN, Bot M, Scheffer PG (2015). Is depression associated with increased oxidative stress? A systematic review and meta-analysis. Psychoneuroendocrinology.

[57] Pan A, Keum N, Okereke OI (2012). Bidirectional association between depression and metabolic syndrome: a systematic review and meta-analysis of epidemiological studies. Diabetes Care.

[58] Zhuang Q-S, Shen L, Ji H-F (2017). Quantitative assessment of the bidirectional relationships between diabetes and depression. Oncotarget.

[59] Jannasch F, Kröger J, Schulze MB (2017). Dietary patterns and type 2 diabetes: a systematic literature review and meta-analysis of prospective studies. J Nutr.

[60] Godos J, Zappalà G, Bernardini S (2017). Adherence to the Mediterranean diet is inversely associated with metabolic syndrome occurrence: a meta-analysis of observational studies. Int J Food Sci Nutr.

[61] Joinson C, Kounali D, Lewis G (2017). Family socioeconomic position in early life and onset of depressive symptoms and depression: a prospective cohort study. Soc Psychiatry Psychiatr Epidemiol.

[62] Weich S, Lewis G (1998). Poverty, unemployment, and common mental disorders: population based cohort study. BMJ.

[63] Appleton KM, Woodside JV, Yarnell JWG (2007). Depressed mood and dietary fish intake: direct relationship or indirect relationship as a result of diet and lifestyle?. J Affect Disord.

