# Supplementary material for: Association of food groups with depression and anxiety disorders
Source: Eur J Nutr. 2019 Apr 3;59(2):767–78. doi: 10.1007/s00394-019-01943-4 (PMC7058560; doi:10.1007/s00394-019-01943-4)
Supplement: Supplementary file 1 — Supplementary material 1 (DOCX 93 KB) [file 394_2019_1943_MOESM1_ESM.docx]

Energy (kcal/day)

MDS Score

**Food Groups**

Non-refined grains

Vegetables

Fruit

Fish

Olive oil

Red and processed meat

Potatoes

Legumes and Soya

High fat dairy

Poultry

Heavy-Drinker

Non-Drinker

**Supplementary Fig 1** The association between standardised food group residuals with the standardised severity of depression (IDS), anxiety (BAI) and FEAR

*Significant after correction for multiple testing = Negatively scored in MDS = Positively scored in MDS

Adjusted for age, sex, education (yrs)

**β**

**β**

**β**

**Supplementary Fig 2** The association between standardized food group residuals with current depression and remitted depression compared to controls

*Significant after correction for multiple testing = Negatively scored in MDS = Positively scored in MDS

Adjusted for age, sex, education (yrs)

Energy (kcal/day)

MDS Score

**Food Groups**

Non-refined grains

Vegetables

Fruit

Fish

Olive oil

Red and processed meat

Potatoes

Legumes and Soya

High fat dairy

Poultry

Heavy-Drinker

Non-Drinker

**

*

**Food Groups^1^**

Non-refined grains

Vegetables

Fruit

Fish

Olive oil

Red and processed meat

Potatoes

Legumes and Soya

High fat dairy

Poultry

Heavy-Drinker

Non-Drinker

*

*

*

**β**

**β**

**Supplementary fig 3** The association between standardized food group residuals with the standardized severity of depression (IDS), anxiety (BAI) and FEAR cor**r**ected for all other food groups.

*Significant after correction for multiple testing = Negatively scored in MDS = Positively scored in MDS

^1^Adjusted for age, sex, , education (yrs), partner status physical activity, smoking status

**Food Groups^1^**

Non-refined grains

Vegetables

Fruit

Fish

Olive oil

Red and processed meat

Potatoes

Legumes and Soya

High fat dairy

Poultry

Heavy-Drinker

Non-Drinker

*

*

**Supplementary fig 4** The association between standardized food group residuals with current depression and remitted depression compared to controls corrected for all other food groups

*Significant after correction for multiple testing = Negatively scored in MDS = Positively scored in MDS

^1^Adjusted for age, sex, , education (yrs), partner status physical activity, smoking status
