# Supplementary material for: Association of food groups with depression and anxiety disorders
Source: Eur J Nutr. 2019 Apr 3;59(2):767–78. doi: 10.1007/s00394-019-01943-4 (PMC7058560; doi:10.1007/s00394-019-01943-4)
Supplement: Supplementary file 2 — Supplementary material 2 (DOCX 15 KB) [file 394_2019_1943_MOESM2_ESM.docx]

| **Supplementary table 1** Effect sizes (Pearsons correlation coefficient) for the association between standardized food group residuals, energy and MDS with the standardised severity of depression (IDS), anxiety (BAI) and phobias (FEAR) | | | | | |  |
| --- | --- | --- | --- | --- | --- | --- |
|  | IDS Score (n=1616) |  | BAI (n=1612) |  | FEAR (n=1613) |  |
|  | r |  | r |  | r | |
| Energy (kcal/day) | .06 |  | .07 |  | -.08 | |
| MDS Score | -.11 |  | -.09 |  | .06 | |
| **Food group residuals** |  |  |  |  |  | |
| Non-refined grains | -.10 |  | -.06 |  | -.01 | |
| Vegetables | -.05 |  | -.05 |  | -.10 | |
| Fruit | -.04 |  | .00 |  | -.08 | |
| Fish | -.02 |  | -.03 |  | -.05 | |
| Olive oil | -.02 |  | -.02 |  | -.02 | |
| Red and processed meat^1^ | -.02 |  | -.02 |  | .00 | |
| Potatoes | -.02 |  | -.05 |  | .02 | |
| Legumes and soya | -.02 |  | .00 |  | .03 | |
| High fat dairy^1^ | .01 |  | .02 |  | .00 | |
| Poultry^1^ | .04 |  | .01 |  | .01 | |
| Heavy drinker^1^ | -.02 |  | .00 |  | .00 | |
| Non-Drinker^1^ | .10 |  | .08 |  | .06 | |
| ^1^In the MDS these items are negatively scored, meaning that the direction of association is expected to be the opposite (b<0) of the other food groups | | | | | |  |

| Supplementary table 2: Effect sizes (Cohen’s D) for the association between standardized food group residuals, energy and MDS with current anxiety/depression and remitted depression/anxiety compared to controls. | | |
| --- | --- | --- |
|  | Remitted Depression/anxiety  (compared to controls) | Current depression/anxiety  (compared to control) |
| Energy (kcal/day) | 0.04 | 0.07 |
| MDS Score | -0.01 | -0.10 |
| **Food group residuals** |  |  |
| Non-refined grains | -0.06 | -0.07 |
| Vegetables | 0.04 | -0.04 |
| Fruit | -0.01 | -0.05 |
| Fish | -0.01 | -0.01 |
| Olive oil | 0.04 | 0.00 |
| Red and processed meat^1^ | -0.01 | -0.03 |
| Potatoes | -0.01 | -0.03 |
| Legumes and soya | 0.02 | 0.01 |
| High fat dairy^1^ | -0.03 | -0.01 |
| Poultry^1^ | 0.04 | 0.04 |
| Heavy drinker^1^ | -0.06 | -0.03 |
| Non-drinker ^1^ | 0.03 | 0.09 |
| ^1^In the MDS these items are negatively scored, meaning that the direction of association is expected to be the opposite (b<0) of the other food groups | | |
